# Ammonium Polyphosphate Intercalated Layered Double Hydroxide and Zinc Borate as Highly Efficient Flame Retardant Nanofillers for Polypropylene

**DOI:** 10.3390/polym10101114

**Published:** 2018-10-09

**Authors:** Yanshan Gao, Qiang Wang, Weiran Lin

**Affiliations:** 1 Beijing Key Lab for Source Control Technology of Water Pollution, Beijing Forestry University, 35 Qinghua East Road, Haidian District, Beijing 100083, China; yanshan_gao@bjfu.edu.cn; 2 Beijing Research Institute of Chemical Industry, SINOPEC, Beijing 100013, China

**Keywords:** flame retardancy, thermal stability, polypropylene, APP-LDH, zinc borate

## Abstract

We found in our previous study that layered double hydroxides (LDHs) which undergo aqueous miscible organic solvent treatment (AMOST) can tune the hydrophobicity surface of LDHs to be hydrophobic, and then the solvent mixing method can be used to prepare polymer/LDH nanocomposites. However, flame retardant property is not very high if LDHs are only used. In this present work, ammonium polyphosphate (APP) intercalated LDHs and zinc borate (ZB) was incorporated into a polypropylene (PP) matrix using the solvent mixing method. The structures, morphologies, and performance of the composites were characterized carefully. The peak heat release rate (PHRR) reduction of PP containing 10 and 20 wt % APP-LDH reached 27% and 55%, respectively, which increased up to 63% compared with PP/CO_3_-LDH. After incorporating 2 wt % ZB in the PP/APP-LDH system, the flame retardant property was further improved. Polypropylene composites with 20 wt % APP-LDH and 2 wt % ZB showed a 58% PHRR reduction. In addition, thermogravimetric analyzer (TGA) results indicated that the addition of APP-LDH and ZB improved the temperature at 50% weight loss (*T*_50%_) and the char formation of the materials significantly.

## 1. Introduction

Polypropylenes (PPs) are involved in a wide range of applications, such as the automotive, home appliance, and construction industries, etc., due to their ease of processing, excellent mechanical, electrical, chemical resistance, and non-toxicity [1]. However, most polymer materials, including PPs, are mainly composed of C, H, and O elements, which are easy to burn and generate smoke and toxic, and harmful gases during their combustion, which is a serious threat to both human bodies and the environment. The increased use of these polymer materials has resulted in a growing awareness of flammability problems. Therefore, the development of suitable flame retardant additives for PPs to delay the production and spreading of flames has attracted much attention [2].

Among the variety of flame retardants, layered double hydroxides (LDHs) have been regarded as effective flame retardants as well as electrocatalysts [3], CO_2_ adsorbents [4], and supercapacitors [5], due to their unique layered structures [6,7,8,9]. Besides, LDHs can improve the thermal stability of polymers as well; a small amount of LDHs can increased the thermostability significantly. However, similar to general inorganic flame retardants, one of the severe problems of LDHs is that their high loading, generally 40~60 wt % can achieve a good degree of flame retardancy when only LDHs are used [10,11], which usually results in poor mechanical and thermal properties of the materials. Hence, in order to overcome this problem, LDHs together with other synergistic flame retardant additives is recommended, such as magnesium hydroxide [12], phosphorus-containing compounds [13,14], carbon-based materials [15], borate-containing materials [16], Ni(OH)_2_ [17], or intumescent flame retardants [18], and so on. Among these, ammonium polyphosphate (APP) shows excellent flame retardant performance because of its lower loadings and cost, as well as good processability [2]. In addition, APP is halogen free, so it will not generate harmful gases during its use, which makes environmentally friendly compared with halogen-containing additives [19]. Some researchers have investigated the synergistic effect between LDH and APP; all composites can reach a UL-94 V-0 rating when incorporating APP in polymer/LDH systems [20,21,22,23]. However, in these studies, APP and LDHs particles were just physically mixed together, and we wondered if APP intercalated LDH (APP-LDHs) could further improve the flame retardant performance of polymers. Therefore, APP-LDHs were synthesized first and the flame retardant and thermal stability behaviors of PP/APP-LDH composites were investigated carefully in this study.

In addition, it is reported that zinc borates (ZBs) combined with LDH can function as a synergistic additive promoting formation of a char residue, as well as acting as a protective layer on the surface of polymers [24,25]. Zinc borates with different chemical formulas are usually used as flame retardant additives [26,27]. In our previous study [28], we demonstrated that a good dispersion of a flame retardant additive in the polymer matrix is also important. However, the ZB particles cannot always be dispersed well in polymers. In this contribution, we report the synthesis of one kind of Zn_3_B_10_O_18_·14H_2_O morphology—a combination of rods and particles by solution method—and turn its surface hydrophobic so that it can be uniformly dispersed in the polymer matrix. 

In this work, we take advantage of the layered structure of LDHs and the superior flame retardant performance of APP, synthesizing APP intercalated LDHs and investigate both the fire resistance and thermal stability of APP-LDH. On the basis of this, the synergistic effect of LDHs and zinc borate is considered as well.

## 2. Materials and Methods

### 2.1. Materials

Materials were procured as follows: polypropylene (average molecular weight ca. 300,000, The Dow Chemical Company, Midland, MI, USA), Mg(NO_3_)_2_·6H_2_O (AR, Sinopharm Chemical Reagent Co., Ltd., Shanghai, China), Al(NO_3_)_3_·9H_2_O (AR, Sinopharm Chemical Reagent Co., Ltd., Shanghai, China), APP (Adamas-beta Company, Basel, Switzerland), Na_2_B_4_O_7_·10H_2_O (AR, Sinopharm Chemical Reagent Co., Ltd., Shanghai, China), ZnSO_4_·7H_2_O (AR, Sinopharm Chemical Reagent Co., Ltd., Shanghai, China), H_3_BO_3_ (AR, Sinopharm Chemical Reagent Co., Ltd., Shanghai, China), acetone (AR, Beijing Chemical Works, Beijing, China), sodium hydroxide (AR, Sinopharm Chemical Reagent Co., Ltd., Shanghai, China), xylene (AR, Beijing Chemical Works, Beijing, China), and hexane (AR, Sinopharm Chemical Reagent Co., Ltd., Shanghai, China).

### 2.2. Synthesis of Aqueous Miscible Organic (AMO)-LDH

Mg_3_Al-APP LDH was synthesized using a traditional hydrothermal approach. A solution containing 9.60 g Mg(NO_3_)_2_·6H_2_O and 4.70 g Al(NO_3_)_3_·9H_2_O in 50 mL deionized water and NaOH (4 M) was added into an APP solution (25 g APP in 50 mL deionized water), the pH of the system was ~10. The obtained mixture slurry was sealed in an autoclave and reacted at 100 °C for 24 h. After the reaction, the slurry was centrifuged and washed with water until its pH was neutral condition. Then it was treated with acetone to tune the LDH surface from hydrophilic to hydrophobic. For the PP/APP-LDH nanocomposites, the resultant AMO-LDH was directly used without drying. Mg_3_Al-CO_3_ LDH was synthesized similarly. 

### 2.3. Synthesis of Zinc Borate

Zinc Borate with uniform morphology was prepared at 90 °C using Na_2_B_4_O_7_·10H_2_O, ZnSO_4_·7H_2_O and H_3_BO_3_ as raw materials. In detail, the mixture of 19.07 g of Na_2_B_4_O_7_·10H_2_O, 6.18 g of H_3_BO_3_, and 50 mL of deionized water was stirred at 70 °C. Then 14.38 g of ZnSO_4_·7H_2_O was added into the mixture solution and further stirred for 8 h at 90 °C. The obtained white precipitates were similarly treated with LDH.

### 2.4. Preparation of PP/APP-LDH and PP/APP-LDH/ZB Nanocomposites

The solvent mixing method was used to synthesize PP/APP-LDH nanocomposites. In detail, a certain amount of PP and APP-LDH (total amount 5 g, the LDH loading in PP was 10 and 20 wt %, respectively) was added into 100 mL xylene. Refluxing at ~140 °C for at least 2 h to obtain dissolved PP solution with highly dispersion of LDH particles. Then the hot solution was poured into 100 mL hexane. After being filtered and dried, the PP/APP-LDH nanocomposites were obtained. Polypropylene/APP-LDH/ZB nanocomposites were prepared in the same way.

### 2.5. Characterization of Samples

X-ray diffraction (XRD) results were performed on a Shimadzu XRD–7000S instrument (Cu Kα radiation, the accelerating voltage 40 kV, current 30 mA (λ = 1.542 Å), 2θ degree from 2° to 70°, scanning rate 5° min^−1^, Kyoto, Japan). Fourier Transform Infrared Spectroscopy (FT-IR) spectra were recorded on a Bruker VERTEX 70 FT-IR spectrophotometer (400–4000 cm^−1^, 100 scans with a resolution of 4 cm^−1^, Munich, Germany). Scanning electron microscopy-energy dispersive spectrometer (SEM-EDS) analyses were recorded on a Hitachi SU–8010 machine (accelerating voltage of 5.0 kV, Tokyo, Japan). ^27^Al NMR analysis was performed on a Bruker Advance 500 spectrometer (Munich, Germany) at a resonance frequency of 130.44 MHz with a 4 mm triple-resonance magic angle spinning (MAS) probe; the sample spinning rate was 10 kHz. Differential scanning calorimetry (DSC) analyses were performed using a Shimadzu TA-60WS instrument (Kyoto, Japan), heating rate was 10 °C·min^−1^, nitrogen flow rate was ca. 50 mL·min^−1^.

### 2.6. Thermal Stability and Flammability Properties

The thermal stabilities were evaluated using a TGA Q50 machine (TA Instruments, New Castle, DE, USA), the heating rate was 10 °C·min^−1^, and the air flow rate was 20 mL min^−1^. Experiments were undertaken from 20 to 600 °C. The flammability performance was investigated using a microscale combustion calorimeter (MCC-2, Govmark, Farmingdale, NY, USA), with sample mass of 5 mg and heating rate of 1 °C s^−1^, and nitrogen flowing of 80 cm^3^ min^−1^. Experiments were undertaken from room temperature to 700 °C.

## 3. Results

### 3.1. Characterization of LDHs and Zinc Borate 

**Figure 1:**
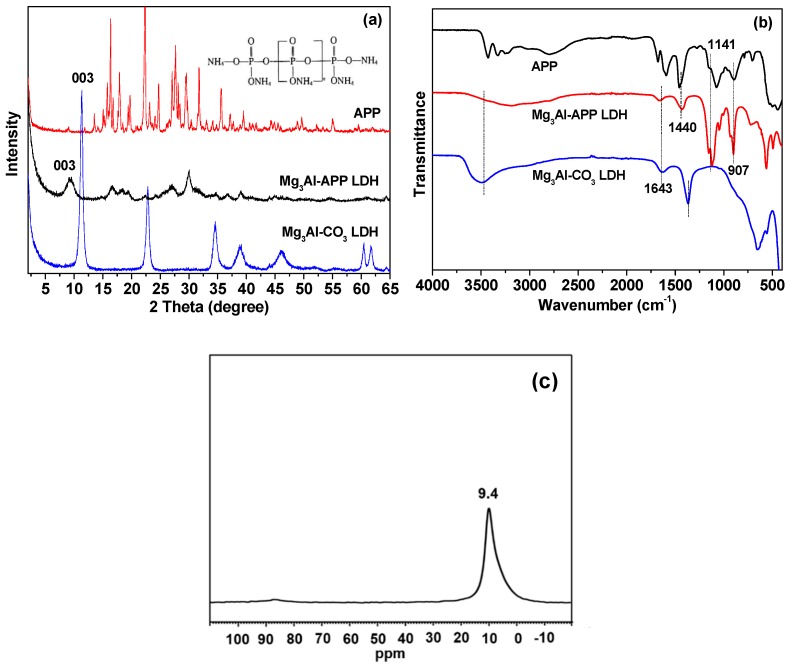
(**a**) X-ray diffraction (XRD) patterns of APP, Mg_3_Al-CO_3_ LDH, and Mg_3_Al-APP LDH; (**b**) FT–IR spectrum of APP, Mg_3_Al-CO_3_ LDH, and Mg_3_Al-APP LDH; and (**c**) ^27^Al solid NMR spectra of LDH.

Figure 1a presents the XRD patterns of APP, Mg_3_Al-CO_3_ LDH, and Mg_3_Al-APP LDH. Mg_3_Al-CO_3_ LDH exhibited the typical reflections of LDHs; the sharp basal reflection (00*l*) harmonics at relatively low 2θ angles indicated a well-crystallized structure of LDH. The characteristic Bragg reflection of (003) was observed at 11.36°, corresponding to a 0.78 nm interlayer spacing. For APP-LDH, the (003) diffraction peak moved to a lower angle (2θ = 9.34°), with an interlayer distance of 0.93 nm, which can be attributed to the intercalation of the relatively larger molecule of APP in the LDH interlayers. Nevertheless, Bragg reflections with low intensity were observed in Mg_3_Al-APP LDH. This is perhaps because the APP-LDH become more amorphous, and thus there is insufficient coherence along the platelet stacking axis (*c*-axis) to observe any high intensity (00*l*) Bragg reflection [29].

In order to have a better understanding of the LDH structure, ^27^Al solid state NMR analysis was performed, as shown in Figure 1c. The NMR spectra of LDH nanoparticles exhibited a single resonance near 9.4 ppm, which can be assigned to octahedrally coordinated Al [30]. The result confirmed that the LDH structure was formed by metals coordinated to six hydroxyl groups with octahedral geometry.

Fourier transform infrared spectroscopy spectra of APP, Mg_3_Al-CO_3_ LDH, and Mg_3_Al-APP LDH are displayed in Figure 1b. A broad band around 3400–3500 cm^−1^ was due to the OH stretching of water molecules, and the bending vibration around 1643 cm^−1^ was also due to the interlayer of H_2_O. The adsorption peak at 562 cm^−1^ belongs to MO, OMO, and MOM lattice vibrations (M represents Mg and Al). The band at 1365 cm^−1^ is attributed to the interlayer of CO3^2−^. Besides, the bands at 1141, 907, and 1440 cm^−1^ are attributed to the vibrations of P–O and N–H group, respectively, demonstrating the presence of phosphate in the obtained APP-LDH nanoparticles [29]. The spectra results suggest a successful intercalation of APP molecules into the interlayers of LDH and that they were not decomposed.

**Figure 2:**
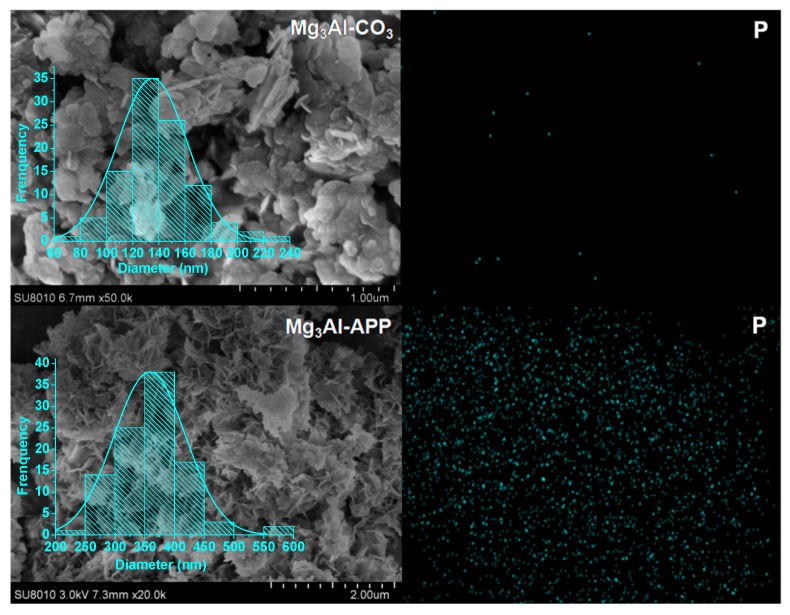
SEM images of Mg_3_Al-CO_3_ and Mg_3_Al-APP LDH and corresponding EDS mapping of phosphorus.

Figure 2 presents the SEM-EDS images of Mg_3_Al-CO_3_ LDH and Mg_3_Al-APP LDH. Both LDHs have formed plate-like nanoparticles with plate-like shapes with a diameter around 100~300 nm, according to the SEM images, and normal distribution of particles, which is consistent with previous studies [9,31]. However, the particle diameters of APP intercalated with LDH were a little larger than the carbonate intercalated with LDH. The EDS mapping indicates the presence of P elements in the Mg_3_Al-APP LDH, whereas almost no P elements were found in the Mg_3_Al-CO_3_ LDH, further confirming that APP molecule existed in the LDH interlayers. The EDS results are in agreement with both XRD and FT-IR results.

**Figure 3:**
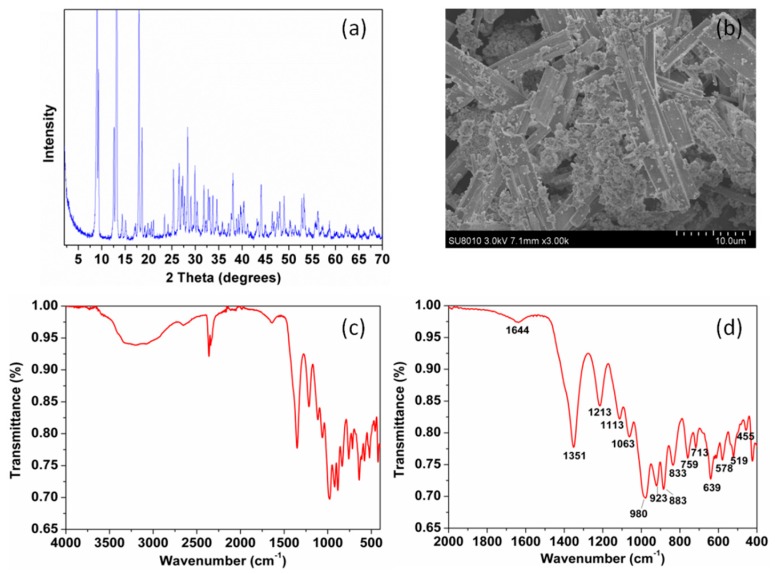
(**a**) XRD patterns, (**b**) SEM images, (**c**) FT-IR spectrum, and (**d**) enlarged FT-IR spectrum of zinc borate.

Figure 3a represents the XRD patterns of the ZB. The XRD results were consistent with the pure phase of Zn_3_B_10_O_18_·14H_2_O (File No. 32-1461), indicating that the ZB was synthesized successfully. The FT-IR spectra of ZB exhibited some bands and can be assigned as follows (Figure 3c,d) [32,33,34]. The band at 3300–3400 cm^−1^ is the O–H stretching while the adsorption peaks at 2655 cm^−1^ is because of hydrogen band. The band at 1644 cm^−1^ can be attributed to the H–O–H from crystalline water. The characteristic bands at 1351 and 923 cm^−1^ are the asymmetric and symmetric stretching of B_(3)_–O (means three coordinate boron). The peak at 1213 cm^−1^ represent the bending of B–O–H. The peaks at 1113 cm^−1^, 833 cm^−1^, and 759 cm^−1^ are the stretching of B_(4)_–O (means four coordinate boron). The band at 639 cm^−1^, 519 cm^−1^, and 578 cm^−1^ are derived from B_(3)_–O. The peak at 455 cm^−1^ is due to the B_(4)_–O bending. Fourier transform infrared spectroscopy results further confirmed the successful synthesis of ZB. As seen in Figure 3b, the morphologies of the materials are combination of rods (about 1–3 × 10 μm) and particles (diameter less than 1 μm). 

### 3.2. Characterization of PP/APP-LDH and PP/APP-LDH/ZB Nanocomposites

**Figure 4:**
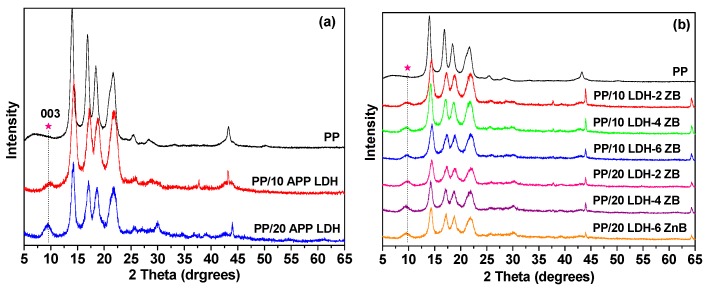
XRD patterns of PP, (**a**) PP/APP-LDH, and (**b**) PP/APP-LDH/ZB nanocomposites.

Figure 4 presents the XRD results of pure PP and PP nanocomposites containing LDH and ZB. Pure PP has a semi-crystalline structure with several main reflections at 14–23°. For both PP/10 APP-LDH and PP/20 APP-LDH nanocomposites, a reflection at 2θ = 9.3° can be found in Figure 4a, which correspond to the (003) reflection of APP-LDH. This results indicate that the dispersed LDH retains a layer structure after solvent mixing with PP. The (003) peak also increased with the increasing of LDH addition amount. However, when the PP/LDH nanocomposites were prepared with 2–6 wt % ZB (Figure 4b), only the typical peaks of PP and LDH can be seen, suggesting that the ZB was dispersed in the PP matrix amorphously.

**Figure 5:**
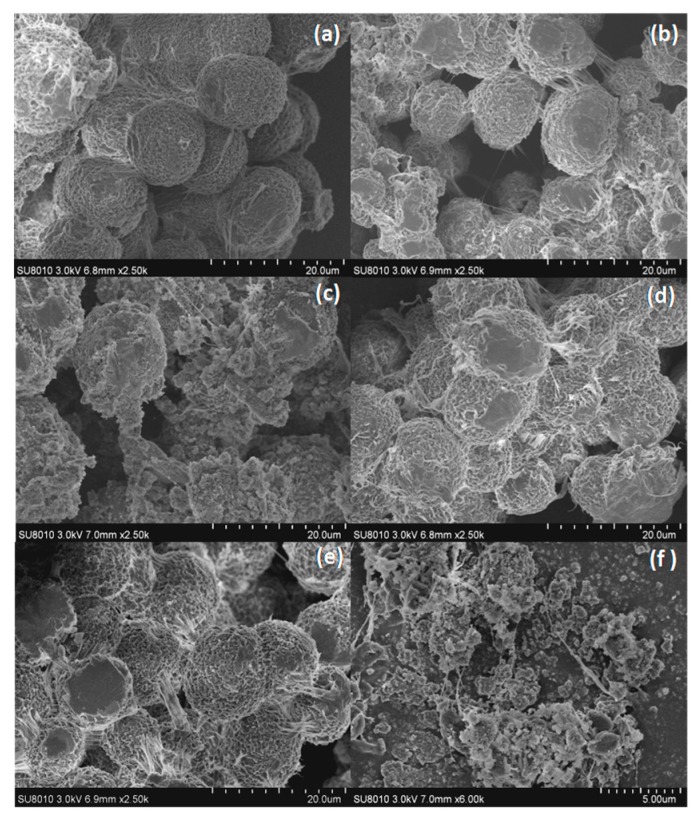
SEM results of (**a**) PP, (**b**) PP/10% APP-LDH, (**c**) PP/20% APP-LDH, (**d**) PP/2% ZB, (**e**) PP/10% APP-LDH/2% ZB, and (**f**) PP/20% APP-LDH/2% ZB nanocomposites.

Scanning electron micrographs of the samples are shown in Figure 5. Pure PP shows a spherical morphology with a very smooth surface (Figure 5a). After adding 10–20 wt % LDH, the PP/LDH nanocomposites became rough, especially with 20 wt % LDH, there were imperfections in the spherical structure (Figure 5c). However, nearly no LDH nanoparticles can be seen on PP surface, indicating that the LDH nanoparticles can be excellently dispersed in PP matrix by acetone modification. The PP/2% ZB nanocomposites were very similar with PP in terms of their morphologies and sizes. When combining 10% LDHs with 2% ZB together, both LDH particles and ZB can rarely be observed on the surface of the spherical-like PP; however, the spherical structure was destroyed when the LDH increased to 20%, this was due to the high loading of LDH.

### 3.3. Thermal and Flammable Properties of PP-Based Nanocomposites

**Figure 6:**
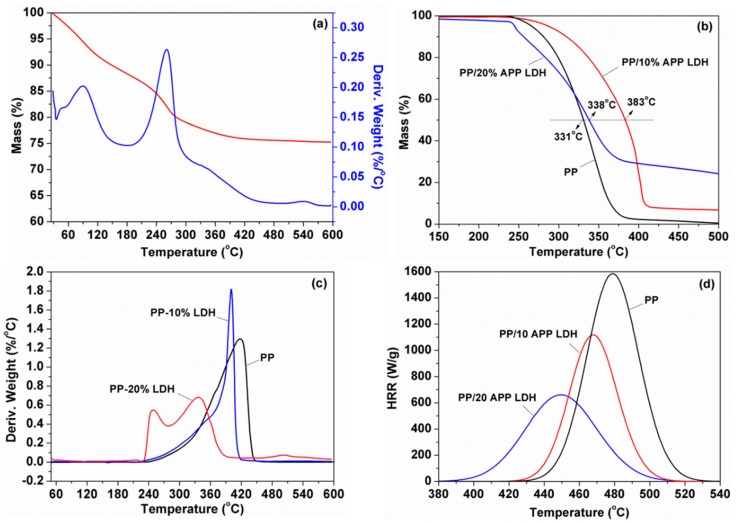
(**a**) Thermogravimetric-differential thermal gravity (TGA-DTG) curve of APP-LDH, (**b**) TGA, and (**c**) DTG curves of pure PP and PP/APP-LDH nanocomposites, and (**d**) microscale combustion calorimeter (MCC) analyses of pure PP and PP/APP-LDH nanocomposites.

In order to obtain a composite with perfect properties, it was necessary to understand the thermal decomposition [35,36]. The typical thermogravimetric analyzer (TGA) curves of APP-LDH and PP/LDH nanocomposites are presented in Figure 6. The relative thermal stability of the samples was usually evaluated by the temperature at 10% weight loss (*T*_10%_), the temperature at 50% weight loss (*T*_50%_), and the char residual percentage at 600 °C, as listed in Table 1. In contrast to pure PP, the *T*_10%_ of PP/10% APP-LDH nanocomposites increased by 30 °C; however, the PP nanocomposites with 20% APP-LDH exhibited a reduction in *T*_10%_, which was probably attributed to the decomposition of the LDH particles, as shown in Figure 6a. Besides, when the loading of LDH increased, the addition of APP-LDH promoted and catalyzed the degradation of PP to a small extent in the initial stage, thus the *T*_10%_ decreased. The char layer catalyzed by APP-LDH slowed down the heat release rate upon the ignition of PP, and thus protected the inside polymer matrix from being degraded by the flame. As to the *T*_50%_, both PP nanocomposites increased no matter with 10% or 20% LDH loading. However, the *T*_50%_ of PP/10% APP-LDH increased by 52 °C, while it only increased 7 °C for PP/20% APP-LDH (Figure 6b). This result indicated that addition of LDH particles could enhance the thermal stability of polymers significantly, but it would decrease with a high loading. Therefore, it is necessary to reduce the additives loading in polymers under the premise of highly efficient flame retardant performance. In addition, only 0.7 wt % char residue was obtained for pure PP. The addition of LDHs also improved the char yield to 7.0 and 24.1 wt % from 0.7 wt % for PP/10% APP-LDH and PP/20% APP-LDH, respectively.

To confirm the function of the char forming, the theoretical char residue of PP/APP-LDH was calculated by linear combination, as shown in Table 1 [37]. The experimental and theoretical char yield for PP/20% APP-LDH nanocomposites are 24.1 and 15.6 wt %, respectively. It is obvious that the calculated char yield was much lower than the measured one, which further suggested that the addition of APP-LDH enhanced the char formation. 

Figure 6d displays the MCC results of PP and PP/APP-LDH nanocomposites. It can be observed that PP burned rapidly after ignition, for which the peak heat release rate (PHRR) value was 1585 kWm^−2^. As expected, incorporating 10 or 20 wt % APP-LDH into PP significantly decreased the PHRR value to 1154 and 707 kWm^−2^, respectively, for the which reduction was 27% and 55%. Besides, both the total heat release (THR) and heat release capacity (HRC) values decreased significantly (Table 2).

## 4. Discussion

**Figure 7:**
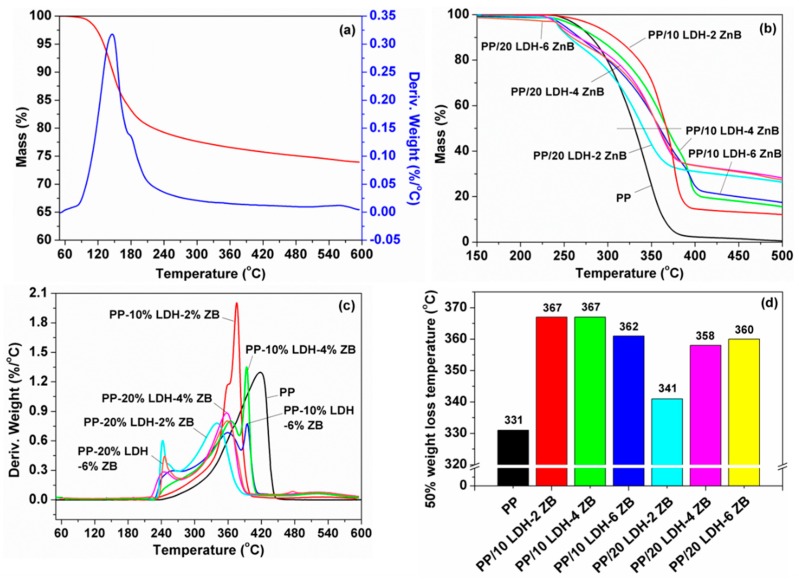
(**a**) Thermogravimetric-Differential thermal gravity (TGA-DTG) curve of ZB, (**b**) TGA and (**c**) DTG curves of pure PP and PP/APP-LDH/ZB nanocomposites, and (**d**) 50% weight loss temperature of all the samples.

In order to further increase the flame retardant properties of PP polymers, at the same time, to not affect the thermal stability of the composites caused by the high additives loading, ZB was added to PP/LDH systems and the thermal stability and flame retardant performance was studied systematically. Thermogravimetric analyzer (TGA) analyses for PP and PP/LDH/ZB composites are shown in Figure 7 and the corresponding results are listed in Table 1. After incorporation of ZB to PP/LDH systems, The *T*_50%_ increased significantly when compared to pure PP, especially for PP/LDH/ZB composites with 10 wt % LDH. The increased value was slightly lower than PP/LDH composites without ZB (52 °C), but the value of *T*_50%_ increased by at least 30 °C no matter with 2, 4, or 6 wt % ZB together. When the LDH loading increased to 20 wt % LDH, the *T*_0.5_ of PP/LDH/ZB composites increased 10, 27, and 29 °C, respectively. Differential scanning calorimetry (DSC) analysis of pure PP and PP/LDH/ZB composites was studied as well. As shown in Figure S1, for pure PP, there is only one endothermic peak located at around 175 °C, corresponding to the melting points of PP polymer. After addition of LDH/ZB, another endothermic peak at around 225~233 °C can be observed, which can be attributed to the existence of LDH. The first degradation of ZB was around 170 °C according to TGA and DTG results, which was close to pure PP and the melting point of ZB was very high, laying above the upper temperature of the DSC experiment, so there were no more peaks for PP/LDH/ZB composites. In addition, the experimental char yield of PP/LDH/ZB composites was much higher than the theoretical one, indicating the LDH/ZB additives promoted the formation of char during combustion. Moreover, it is worth emphasizing that after adding ZB to PP/LDH system, the experimental char yield also increased, suggesting the addition of ZB further accelerated the char residue.

**Figure 8:**
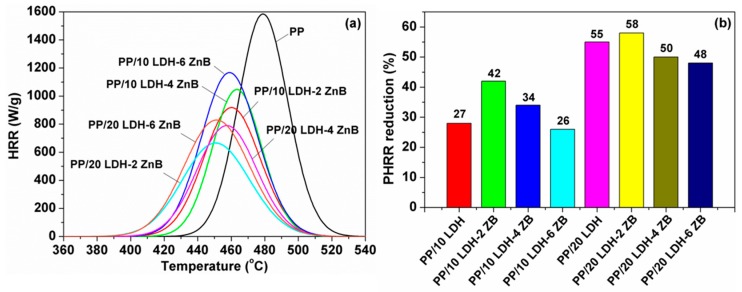
(**a**) HRR curves and (**b**) PHRR reduction of PP and PP/APP-LDH/ZB nanocomposites prepared.

For the flammability properties, pure PP burnt easily with a high PHRR value of 1585 W g^−1^, after incorporation of 10 wt % and 20 wt % APP-LDH, and the PHRR value decreased by 27% and 55%, respectively (Table 2), which was much higher than carbonate intercalated LDH. However, on the basis of this, the addition of ZB further increased the flame retardant factor of PP. Figure 8 represents the HRR curves of PP composites and the corresponding results are listed in Table 2 as well. When adding 2 wt % ZB, the PHRR value of PP/LDH/ZB composites with 10 wt % LDH loading was 918 Wg^−1^, which was reduced by 42% compared to pure PP, which decreased by 20% compared to the PP/LDH composites. However, when loading more ZB, such as 4 or 6 wt %, the PHRR was slightly lower than 2 wt % loading. Therefore, it can be concluded that only 2 wt % ZB could result in better flame retardant performance, and excessive ZB may decrease the flame retardancy. Similar results were obtained for the PP/LDH/ZB composites with 20 wt % LDH. The mechanism for ZB to improve the flame retardancy of polymer can be attributed to the glassy layer formed by it which can protect the inner polymer materials from further burning [38].

**Figure 9:**
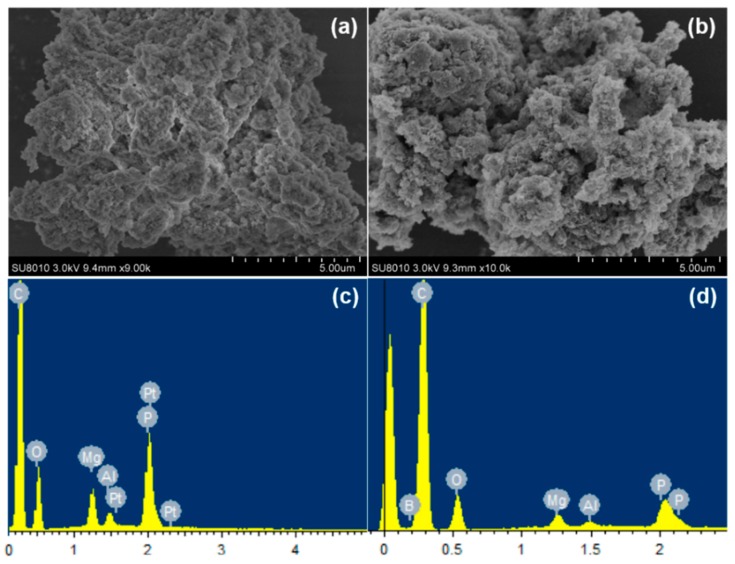
SEM-EDS images of (**a**,**c**) PP/20 w t% APP-LDHs and (**b**,**d**) PP/20 wt % APP-LDHs/2 wt % ZB composites char residue after combustion.

In order to study the char residue morphologies of PP/LDH and PP/LDH/ZB composites, SEM-EDS analyses of the residue char is showed in Figure 9. During combustion, APP in the LDH interlayers will work as a vigorous dehydrating agent, which can react with PP and decompose to form carbides, phosphorus oxides or phosphoric acid covering the surface of the composites (Figure 9a,c). On the one hand, it can prevent the inner PP from further thermal degradation as well as stop the combustibles produced by the degradation contacting to the surface of PP. On the other hand, the formed layer can block the contact of outside flammable gases such as oxygen with polymers, delaying the thermal decomposition and burning rate of the materials.

After incorporation of ZB to PP/APP-LDH composites, the carbon layer formed by thermal decomposition of APP can react with the glassy substance formed by ZB decomposition, which covers the surface of PP composites, making the char layer puffy and compact (Figure 9b). This is because ZB belongs to a low-melting vitreous humour, which will form a viscous substance when heated, and further seals the carbon layer formed by burning, blocking the heat exchange behavior between the material and the outside, thereby slowing the burning rate of the material. Moreover, the thermal stability of the carbon layer will be enhanced due to the presence of a zinc-containing compound, and thus reduce the decomposition rate of the materials greatly. From EDS analyses, except phosphorus, boron also can be observed in PP/APP-LDH/ZB composites, suggesting the existence of ZB. However, because of the very low loading of ZB (2 wt %), zinc did not exist in the sample. In a word, it is believed that ZB combined with LDH can function as a synergistic additive to promote the formation of char residue to protect the polymers. 

**Figure 10:**
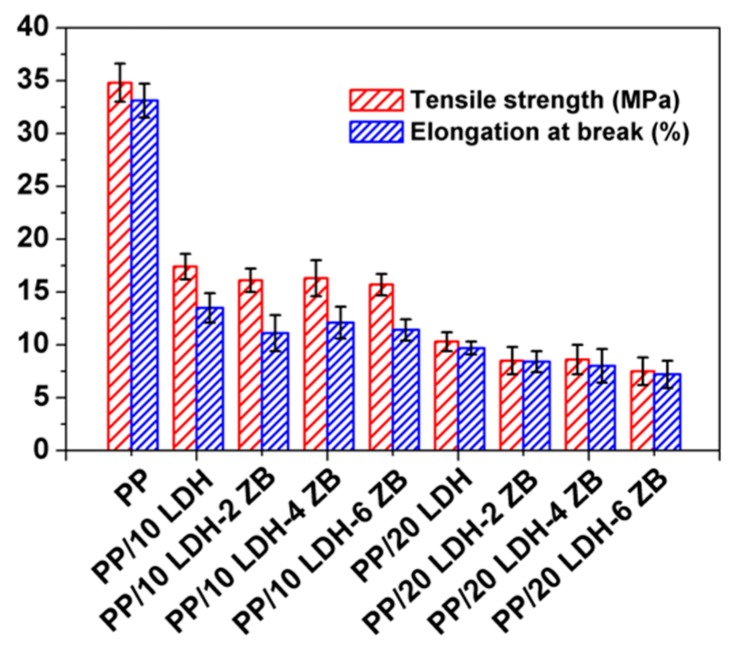
Mechanical properties of PP and PP/LDH nanocomposites.

Since inorganic nanofillers can affect the mechanical properties, especially when the additives loading is high, the tensile strength (TS) and elongation at break (EB) was studied, and the results are shown in Figure 10. The TS and EB of pure PP was 34.8 MPa and 33.1%, respectively. After adding LDH nanofillers, both TS and EB decreased significantly. However, the mechanical properties of PP/LDH/ZB nanocomposites have not decreased very much while the flame retardant performance improved as expected. Therefore, the addition of ZB is better in flame retardancy as well as in maintaining the mechanical properties of PP/LDH nanocomposites.

## 5. Conclusions

In this contribution, the PP/APP-LDH and PP/APP-LDH/ZB nanocomposites were synthesized by solvent mixing method successfully. Both APP-LDH and ZB dispersed well within the PP matrix after aqueous miscible organic solvent treatment (AMOST). Microscale combustion calorimeter (MCC) and TGA results displayed that the APP-LDH had an outstanding flame retardant and thermal stability property for PP. With 10 and 20 wt % addition, the PHRR reduction reached to 27% and 55%, and the *T*_50%_ increased by 52 °C and 7 °C, respectively. We also found that addition of a very small amount of ZB can enhance the flame retardancy and thermal stability further. This is because ZB can form a viscous substance and make the carbon layer more sealed. Therefore, it is a significant attempt to improve the flame retardant and thermal stability properties of PP by using halogen free additives.

## Figures and Tables

**Table 1:** Summary of TGA results for PP, PP/APP-LDH, and PP/APP-LDH/ZB nanocomposites *^a^*.

Samples	*T*_10%_ (^o^C)	Δ*T*_10%_ (°C)	*T*_50%_ (°C)	Δ*T*_50%_ (°C)	Char_(E)_ *^b^* (wt %)	Char_(T)_ *^b^* (wt %)
PP	280	NA	331	NA	0.7	-
PP/10% APP-LDH	310	30	383	52	7.0	6.8
PP/20% APP-LDH	257	−23	336	7	24.1	15.6
PP/10% APP LDH-2% ZB	308	28	367	36	12.0	9.6
PP/10% APP LDH-4% ZB	288	8	367	36	15.4	11.1
PP/10% APP LDH-6% ZB	281	1	362	31	17.6	12.6
PP/20% APP LDH-2% ZB	255	−25	341	10	26.3	17.1
PP/20% APP LDH-4% ZB	267	−13	358	27	28.3	18.5
PP/20% APP LDH-6% ZB	259	−21	360	29	27.4	20.0

*^a^T*_10%_ = temperature at 10% mass loss, *T*_50%_ = temperature at 50% mass loss; Δ*T* = difference between pure PP and the nanocomposites. *^b^* Char_(E)_ = experimental char yield; Char_(T)_ = theoretical char yield.

**Table 2:** Summary of microscale combustion calorimeter (MCC) results for PP, PP/APP-LDH, and PP/APP-LDH/ZB nanocomposites *^a^*.

Samples	PHRR (Wg^−1^)	Reduction (%)	THR (kJg^−1^)	*T*_max_ (°C)	HRC (Jg^−1^K^−1^)
PP	1585	NA	47.6	479	1163
PP/10% CO_3_-LDH	1422	10	43	481.8	1071
PP/20% CO_3_-LDH	1099	31	37.7	484.5	822
PP/10% APP LDH	1154	27	42.5	468	1097
PP/20% APP LDH	707	55	34.0	455	661
PP/10% APP LDH-2% ZB	918	42	40.3	460	869
PP/10% APP LDH-4% ZB	1047	34	40.0	463	990
PP/10% APP LDH-6% ZB	1167	26	37.3	459	880
PP/20% APP LDH-2% ZB	668	58	34.5	451	636
PP/20% APP LDH-4% ZB	790	50	35.0	458	750
PP/20% APP LDH-6% ZB	830	48	29.7	452	619

*^a^* PHRR = peak heat release rate; THR = total heat release; *T*_max_ = temperature at maximum pyrolysis rate; HRC = heat release capacity.

## Supplementary Materials

The following are available online at http://www.mdpi.com/2073-4360/10/10/1114/s1, Figure S1: DSC curves (exo up) of the pure PP and PP/LDH/ZB composites.
