# Supplementary material for: Ammonium Polyphosphate Intercalated Layered Double Hydroxide and Zinc Borate as Highly Efficient Flame Retardant Nanofillers for Polypropylene
Source: Polymers (Basel). 2018 Oct 9;10(10):1114. doi: 10.3390/polym10101114 (PMC6403616; doi:10.3390/polym10101114)
Supplement: Supplementary file 1 [file polymers-10-01114-s001.pdf]

Supplementary Materials: Ammonium Polyphosphate Intercalated Layered Double Hydroxide and Zinc Borate as Highly Efficient Flame Retardant Nanofillers for Polypropylene

Yanshan Gao, Qiang Wang and Weiran Lin

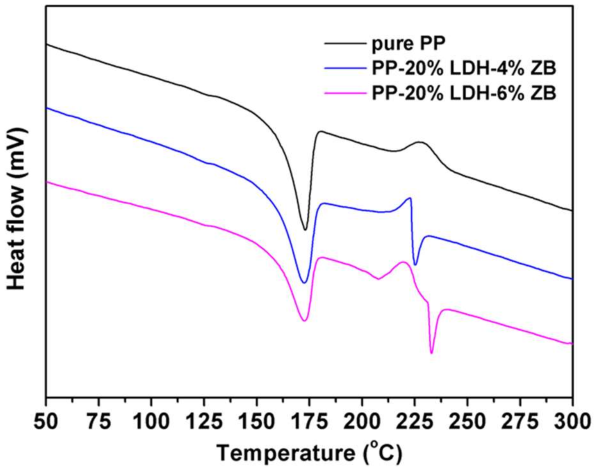

Figure S1. DSC curves (exo up) of the pure PP and PP/LDH/ZB composites.
